# A Systematic Observation of Early Childhood Educators Accompanying Young Children’s Free Play at Emmi Pikler Nursery School: Instrumental Behaviors and Their Relational Value

**DOI:** 10.3389/fpsyg.2020.01731

**Published:** 2020-07-24

**Authors:** Jone Sagastui, Elena Herrán, M. Teresa Anguera

**Affiliations:** ^1^Department of Developmental and Educational Psychology, University of the Basque Country UPV/EHU, Leioa, Spain; ^2^Faculty of Psychology, Institute of Neurosciences, University of Barcelona, Barcelona, Spain

**Keywords:** early childhood, free play, lag sequential analysis, Pikler–Lóczy educational approach, self-determination theory, systematic observation

## Abstract

A significant number of literature documents young children’s innate interest in discovering their surroundings and gradually developing more complex activities and thoughts. It has been demonstrated that when environments support young children’s innate interest and progressive autonomy, they help children acquire a self-determined behavior. However, little is known about the application of this evidence in the daily practice of early childhood educational settings. This study examines Emmi Pikler Nursery School, a center that implements an autonomy-supportive educational approach. We conducted a systematic observation of two experienced Pikler educators while they accompanied young children’s free play. Our objective was to assess: if educators’ instrumental action follows a systematic behavioral sequence, if specific relational behaviors support each instrumental action, and if educators display differentiated intervention levels concerning children’s free play through the adaptation of the relational dimension. We conducted a lag sequential analysis to find behavioral patterns and concurrences between the observed behaviors. Our findings indicate that educators perform systematic action sequences and that each of the instrumental actions of those sequences has a specific relational value. We conclude that educators display three differentiated intervention levels depending on the child and the circumstances of the moment, providing appropriate autonomy support and reinforcing children’s self-determination.

## Introduction

It is well known that play is one of the most important activities for early development and learning. According to Wallon’s (1974) psychogenetic and dialectic theory, the simplest form that precedes play is sensomotor activity, considered the root of thinking, one of the components of intelligence. Its origin was defined as explosive (Wallon, 1980), as it takes the form of an internal need that suddenly wakes up and begins from simple movements, caused by the investigatory or orienting reflex (Pavlov, 1960). It evolves from subjective forms to more objective actions toward the external world (Wallon, 1980): children explore the objects around them, while they try to discover all their interactive possibilities, and through the functional integration of those discoveries, they develop more complex and autonomous activities and thoughts (Wallon, 2008).

In fact, following this author, the specific conduct that a young child performs is a result of a combination of his current competence, the circumstances – physical and human – surrounding it and the child’s capacity to relate himself with those precise circumstances (Wallon, 1980). In this sense, it is important to understand that, from the child’s perspective, they do not actually play, but they live; they live very seriously and get all their functions and emotions involved in every single act that they perform from the moment they are born (White, 1959; Tardos and Szanto-Feder, 2018). Moreover, playful activity gives children the possibility to recreate previously lived experiences (Wallon, 2008), develop their imagination (Vygotsky, 2000), and learn culture-specific behavioral norms (Tomasello, 2000).

In the same line, self-determination theory (Deci and Ryan, 2000) explains the human need to be the origin of their own conduct and the source of that behavior: intrinsic motivation. When it comes to children, they are intrinsically motivated to play from a very early age, as they are innately curious, interested creatures who possess a natural love for learning and a desire to internalize the gained knowledge (Niemiec and Ryan, 2009). Although intrinsic motivation is innate, it is influenced by the three basic psychological needs presented through self-determination theory (Deci, 1975; Deci and Ryan, 2000): autonomy, competence, and relatedness. *Autonomy* refers to the organismic desire to self-organize experience and behavior, and to perform self-endorsed, volitional activities, concordant with one’s integrated sense of self (Deci and Ryan, 2000); it refers to feeling that one is the origin of one’s actions (Joussemet et al., 2005). *Competence* was defined by White (1959) as an energy source that takes the form of an inclination to have an effect on the environment and to attain valued outcomes in it. Finally, although *relatedness* is less central to intrinsic motivation (Deci and Ryan, 2000), this motivation is more likely to flourish in contexts characterized by some sense of secure relatedness (Ryan and La Guardia, 2000).

Thus, intrinsic motivation and, together with it, self-determination require the emergence of the self to develop, which is intimately related to proactivity or own action, accompanied by the need to influence in one’s environment favorably as well as a satisfactory bonding relationship. Even if intrinsic motivation is child-centered, children would not be able to satisfy their psychological needs on their own. Precisely, adults have to ensure that children’s basic psychological needs are satisfied and support their autonomous development (Deci and Ryan, 2000). Specifically, adults’ intervention needs to be particularly delicate when it comes to babies, toddlers, and young children, as their early experiences will determine their future performance. To make sure that young children’s psychological needs are satisfied, adults have to be autonomy-supportive and, consequently, avoid being controlling. Autonomy support means being able to take the other person’s perspective and work from there (Deci, 1995). It facilitates healthy and integrated functioning (Deci and Ryan, 1985, 1991, 2000). Moreover, it has been shown that when environments are autonomy-supportive, they tend to support competence and relatedness needs, too (Ryan and Deci, 2011).

Given that it is the adults’ responsibility to manage the environment that enables child development and to ensure that children relate with it, we have reviewed some studies concerning adults’ role. Many research works have been carried out in schools, given the fact that there cannot be learning without volition (Ryan and Deci, 2011). Their findings demonstrate that, when teachers are autonomy-supportive, elementary school students are more intrinsically motivated, perceive themselves to be more competent, and have higher self-esteem (Deci et al., 1981; Deci, 1995). Furthermore, Ryan and Deci (2000) posited that when teachers harness intrinsic motivation instead of attempting to promote development through external factors, the results in terms of persistence and quality of learning can be profound.

Some other studies have focused on earlier developmental stages due to the importance of social supports for intrinsic motivation to arise (Deci and Ryan, 2000; Ryan and Deci, 2000, 2011). Studies carried out with infants and young children in their family contexts have concluded that parental autonomy support is associated with higher odds of children being globally well adjusted (Joussemet et al., 2005). Maternal autonomy support was found to be a predictor of toddlers’ security of attachment (Whipple et al., 2011) and later executive functioning (Bernier et al., 2010).

This is particularly important as executive function, together with self-regulation skills (Kochanska et al., 2001), are considered vital in today’s world (Yogman et al., 2018) and play has been demonstrated to enable their emergence. Studies have demonstrated that successful educational programs are the ones that support playful learning, in which children are actively engaged in meaningful discovery (Hirsh-Pasek et al., 2009). However, we found fewer investigations focusing on autonomy-supportive educational approaches toward babies, toddlers, and young children at nursery schools. This is especially alarming today, as we live in a society where early schooling (0–3 years) has become a generalized trend, so early childhood education professionals have the challenge of offering babies, toddlers, and young children optimal conditions for their development in those increasingly formalized environments (Hewes, 2014), which is not yet occurring (European Commission, 2019).

In light of this matter, the purpose of the present paper is to scientifically examine an educational setting that has proven to be autonomy-supportive and, concurrently, to suggest it as a recommendable solution to harness babies’, toddlers’, and young children’s healthy development and self-determination.

An early-childhood-oriented educational approach based on the family context but applied and developed in a collective institution – a foster home on its origins and a nursery school nowadays – that has proven to be autonomy-supportive is the one proposed through Pikler–Lóczy education. The four main pillars of this educational approach are (David and Appell, 1986, 2010): the knowledge and recognition of babies’ and toddlers’ early capacities, the establishment of a privileged affective relationship (Falk, 2018a) between each toddler and his or her primary educator, the need to favor each child’s awareness of himself and his surroundings, and the importance of a healthy state, which is the origin as well as a consequence of a proper application of the previous principles. They give a special value to everyday life and recognize daily care routines as the most suitable moments to ensure a healthy and harmonious development as well as an adjusted, progressive, and child-driven learning: caregiving is education (Herrán, 2013).

From Pikler–Lóczy educational approach, when young children have an emotionally stable relationship with their educators, they are happy during the time spent together, but they do not wish to have someone at their side permanently (Szöke, 2016; Falk, 2018b; Tardos, 2018a). During free play episodes, children have a space designed according to their security and exploration needs (Tardos and David, 2018), which arouses a continually renovated pleasure and favors playful activity, if children are developmentally ready and open to it (David and Appell, 1986, 2010; Kálló and Balog, 2013; Tardos, 2014). This kind of activity permits toddlers to live rich experiences, and at the same time, it ensures a psychological space where they can act following their own internal needs (Tardos and David, 2018). By performing their own actions and observing their impacts, babies, toddlers, and young children live an essential learning experience and begin to regulate their conduct autonomously: they learn to learn (Tardos, 2010, 2018a; Tardos and Szanto-Feder, 2018). Therefore, this educational approach ensures that young children have the three psychological needs proposed by self-determination theory (Deci, 1975; Deci and Ryan, 2000) satisfied in the context of the nursery school: autonomy, competence, and relatedness. The privileged affective relationship promotes toddlers’ relatedness in each of their developmental stages and everyday life moments: daily care routines or free play episodes; at the same time, the physical and psychological environment around children is adapted to their needs, so they can attain desired outcomes and have an effect on their surroundings, thus, living their current competence (Pikler, 2018) and finally, they can be autonomous in all circumstances, according to their level.

To ensure that they are autonomy-supportive and adaptive to children, educators have to follow the stages of their development. From this perspective, the essential condition for an efficient educational activity consists of getting to know the children in the group well (Tardos, 2018b). Also, the tool to accomplish this purpose is the observation of the children (Mózes, 2016) during the different moments of everyday life in the nursery school. Educators perform a *participant observation* of children’s action, which translates into a psychological positioning toward them (Mózes, 2016). Precisely, the attention that does not intervene while children are autonomously acting is a basic instrument of Pikler–Lóczy education and sends a significant message to them: “you’re important, what you’re doing is interesting” (Mózes, 2016).

In fact, it was babies’ and toddlers’ observation the tool that gave this educational approach the profound knowledge about early development they nowadays have. The observation of babies’ and toddlers’ development that enabled thorough and comprehensive longitudinal studies carried out during the more than 70 years of favorable experience in the original center where this pedagogy arose – the foster home – (Pikler, 1940, 1968, 1969, 1988, 1998; Pikler and Tardos, 1968; Tardos, 1964, 1967, 1972) contributed to a better understanding about early child capacities. Nowadays, since they opened in 2006, Emmi Pikler Nursery School follows the same principles with toddlers and children living with their families. Specifically, they ensure that each child has a privileged affective relationship with his or her primary educator during the daily care routines and that they have the opportunity to act autonomously and play freely during the rest of the time, under the educators’ attention and adapted help. To ensure the quality of their interventions, educators are trained when they join the institution; they conduct continuous observations and reflect about them together with the pedagogical team of the nursery school (Kelemen, 2016) which makes their professional intervention specific to this model and, therefore, ensures children’s well-being.

Thus, based on their pedagogical positioning, Pikler–Lóczy education would meet the ideas presented through self-determination theory (Deci and Ryan, 2000) due to the autonomy-supportive nature of its educational approach. The management of the daily care routines and the free play episodes is adapted to every child, and the circumstances of the moment, which would ensure children’s basic psychological needs are covered. This justifies our interest in discovering the details that orient the educational activity of Pikler educators.

To deepen in our knowledge about this educational approach, it is basic to get to know the educators’ actions throughout the everyday life of the nursery school, in this case, during children’s free play episodes. The best way to accomplish this goal is by observing their educational activity while they create, maintain, and manage the optimal conditions for spontaneous free play to happen in a natural setting: the regular classroom. For this purpose, we opt to carry out an observational study (Anguera, 2003) due to its numerous possibilities to analyze human interaction in natural contexts, like this one. Observational methodology has a low degree of internal control, as it aims to discover spontaneous behavior in natural settings and, therefore, is considered to be the most suitable for studying it (Sánchez-Algarra and Anguera, 2013). Previous observational studies have demonstrated the existence of structured behavioral patterns in observed educators’ activity during daily care routines such as giving breakfast (Belza et al., 2019a, b) and dressing toddlers (Belasko et al., 2019). Our challenge now is to study the educational activity of educators accompanying children’s free play.

At first sight, while children are playing, educators’ intervention is minimal compared with that in the daily care routines. Children’s autonomous activity seems to be little organized by educators, who come to the play area to tidy up objects or to provide children with new toys, apparently, without following an established order. This study aims to discover if the educational activity of experienced Pikler educators, while they accompany children’s free play, does have an internal structure or if their behavior is an effect of chance. Their educational activity is formed by the educators’ physical or instrumental and human or relational behaviors that intend to guarantee young children’s active role in their activity and their interest, initiative, and progress. That is, what educators do, and how they do it is basic to promote children’s autonomy, competence, and relatedness while they are playing freely.

The purpose of our study was three-fold: firstly, to assess if educators’ instrumental action follows a systematic behavioral sequence, adapted to the circumstances of the moment; secondly, to analyze if each instrumental action by the educator is supported by one or more specific relational behaviors; and thirdly, to discover if the potential sequences of children’s free play accompanying have different levels and characteristics, reflecting an evolving relational adaptation by the educator to the activity of the child at a given time.

We hypothesize that the educational activity of the observed educators is not random but that the instrumental dimension of their educational activity supports children’s free play through intentional and sequential behavior. We also expect that educators adapt the relational dimension of their action to adjust it to the physical and human circumstances of each moment, providing children with proper autonomy support. Consequently, we expect that their educational activity displays differentiated intervention levels, always adapted to the children and their circumstances.

## Materials and Methods

### Design

In this study, we applied a systematic observation to analyze the educational activity of free play accompanying. It is a mixed-methods approach that combines the benefits of qualitative and quantitative methodologies (Anguera and Izquierdo, 2006); as a mixed-methods approach, our proposal consists on organizing qualitative data in a systematized structure (matrix of codes), which is qualitative, but that can be quantitatively analyzed considering the temporal organization of the behaviors. Precisely, observational methodology allows the analysis of spontaneous behavior in natural settings and everyday contexts (Anguera, 2003); therefore, it enables us to obtain rich information by capturing the part of the reality we are interested in (Anguera, 2010).

The observational design (Anguera et al., 2011) of this study was (N/F/M): nomothetic because two educators were observed; follow-up, given that we analyzed sessions recorded in a 3-month time period; and multidimensional because several dimensions of the educational activity were evaluated.

The approach of this study was scientifically rigorous because the observer had a nonparticipatory role in the class and the behaviors observed in the study are fully perceivable (Bakeman and Quera, 2011).

### Participants

The participants of this study were two experienced Pikler educators. The selection of the participants was made between the professional team and the nursery school manager. The chosen educators provided written, informed consent to be video-recorded and for those recordings to be part of research work. The authors’ university’s ethics committee had previously approved the protocol. All procedures were following the ethical standards of this ethics committee and the 2000 Declaration of Helsinki.

We analyzed the educators’ interactive activity toward everything that happened in the play area, inside the regular classroom, during children’s free play episodes. Each of the educators necessarily interacted with her group of children and, also, with different adults who came to the classroom during the direct observation sessions, such as children’s family members or other nursery school staff.

They were both 2- to 3-year-old children groups. Educator 1 had a younger group (average age = 31 months), with a total of 10 children, 5 girls and 5 boys. Educator 2 had older children in her group, 12 in total, 8 girls and 4 boys (average age = 35 months). Written, informed consent was obtained from the parents of minors, too, as they were also video-recorded and involved in the purpose of the study.

### Instruments

#### Observation Instrument

The observation and assessment of educators’ spontaneous behavior in a natural context and other variables around it required the use of an observational tool that was created *ad hoc*. Specifically, we designed a field format (Anguera, 2001, 2003) “*Accompanying free play in Emmi Pikler Nursery School”* (Table 1) that was validated in a previous study (Ormaza, 2016). A field format is a multidimensional, open system that can be codified in multiple, flexible, and self-regulating ways (Anguera, 2003). Its construction begins with the establishment of some general dimensions or criteria related to the observed reality that can unfold more specific subdimensions, under which the observer elaborates a list of the conducts related to each of them, and that achieve the requirement of mutual exclusivity (Anguera, 2003; Anguera et al., 2007).

**TABLE 1:** “Accompanying free play in Emmi Pikler Nursery School” field format.

**Dimension**	**Level I subdimension**	**Level II subdimension**	**Code**
Instrumental behavior	Participants (adults)	Educator	H1
		Other adults	H2
	Participants (children)	Main child(ren)	H3
		Other children	H4
	Physical elements	Space	A1
		Instrumental action	A2
		Materials/toys	A3
		Recipients	A4
Relational behavior	Verbal	Verbal	P1
	Paraverbal	Paraverbal	P2
	Proxemics	Static/alone	P3
		Static/with child	P4
		Movement	P5
	Kinetics	Visual gestures	P6
		Facial gestures	P7
		Hand gestures	P8
		Emblems	P9
		Illustrators	P10
		Regulators	P11

The educational activity during children’s free play combines two dimensions (Wallon, 1985): the *physical/instrumental*, related to the instrumental behaviors that educators perform while children are playing and the physical space where these are developed, and the *interactive/relational*, the one they establish through verbal and nonverbal behavior. Therefore, these two levels were selected as the two main dimensions of the field format. The next step was to specify the subdimensions that would come under each of them.

When it comes to the physical/instrumental aspects that influence educators’ behavior, we decided to define three subdimensions that were later divided into a more specific level. Therefore, the *instrumental* dimension includes three level I subdimensions and eight level II subdimensions. First, the adults present in each particular moment, which includes two level II subdimensions: educator (H1) and other adults (H2). Second, the children involved in each action, including another two level II subdimensions: children in the foreground (H3) and the background (H4) of educators’ attention. Third, the physical elements that define each moment, divided into four level II subdimensions: space (A1), the educators’ instrumental action itself (A2), materials/toys (A3), and recipients (A4). It is a total of three level I subdimensions and eight level II subdimensions, which include 157 behaviors that enabled us to describe the instrumental aspect of the educational activity.

The development of the *relational* dimension was more complex and challenging but yet very important due to our interest in discovering this side of the educators’ behavior. This led us to take the contributions of important theorists into account as a starting point for the construction of the subdimensions under the relational aspect. These define the communicative flow of the educational activity, which accompanies their instrumental action. Specifically, there are 4 level I subdimensions and 11 level II subdimensions regarding educators’ relational behavior: verbal behavior (P1); paraverbal behavior (P2); proxemics (Hall, 1966), divided into static alone (P3), static with child (P4), and movement (P5); and kinetics (Ekman and Friesen, 1969; Ekman, 1976; Poyatos, 1986), divided into visual gestures (P6), face gestures (P7), hand gestures (P8), emblems (P9), illustrators (P10), and regulators (P11). These include a total of 104 behaviors.

The resulting field format can be defined as a very *molecularized* one (Schegloff, 2000) due to the complexity of the observed activity. Specifically, it has unfolded four levels, successively included in the field format: dimensions, level I subdimensions, level II subdimensions, and behaviors. These create a hierarchical system that gives an extremely detailed picture of the educational activity of free play accompanying.

#### Recording Instruments

The sessions were video-recorded in the play area using a SONY DCR-SR37 video camera. Following the principles of the Declaration of Helsinki, participants were informed that they were being filmed. We conducted the systematized recording of the sample using HOISAN program on its 1.6.3.3.6 version (Hernández Mendo et al., 2012).

### Procedure

The sample used in this study – the video-recordings of free play accompanying – was part of wider research and was collected in a 3-month time period. The sessions were delimited respecting two main requisites: within- and between-session consistency (Portell et al., 2015). For this study, a total of 27 sessions were recorded (16 for educator 1 and 11 for educator 2), in which the educators were accompanying children’s free play. The total video-recording time was 4 h 50’ 44”.

Once the data collection was done, we conducted a descriptive recording of all sessions. The main purpose was to divide the video-recordings into behavioral units, which contain the minimum amount of information that has its own meaning (Anguera, 2003). The behavioral units were delimited by the *round-strolls* (Wallon, 2008) that educators take around the play area and in a differentiated way while they tidy up, pick up toys, offer materials, or intervene, depending on the situation of the play area and the child or group of children in each particular moment. We counted with 553 behavioral units on the educators’ free play accompanying (370 for educator 1 and 183 for educator 2).

Then, we proceeded to develop the systematized recording of the behavioral units through the codes specified in the observation instrument. This led us to a codification of the observed reality showing concurrent or simultaneous behaviors that correspond to the subdimensions of the field format, resulting in a record that takes the format of a code matrix. The columns that form the matrix correspond to the 19 level II subdimensions included in both dimensions of the field format. The rows show the codes of the different behaviors – one for each subdimension – that occur simultaneously. Every time that one of the behaviors specified on a row changes, a new row starts, showing the specific behaviors of that particular moment. The succession of the rows that form the matrix illustrates the diachronic development of each session (Anguera, 2010). Its content reveals the enormous complexity of information contained within the human communicative flow (Anguera and Izquierdo, 2006).

#### Quality of the Data

The quality of the data was measured using HOISAN 1.6.3.3.6 program (Hernández-Mendo et al., 2014), by calculating the canonical concordance of Krippendorff (2013) both interobserver and intraobserver. So, 10% of the total sample was recorded by an external researcher, and one of the authors of this study recorded it again a time after recording the whole sample. Both calculations were satisfactory, with a value of 0.83 and 0.84 (0–1).

#### Data Analysis

The quantification is particularly robust in observational methodology, and we opted to apply lag sequential analysis (Bakeman and Gottman, 1997), a technique used to obtain regularities and behavioral patterns in the observed participants. Specifically, it identifies patterns and associations between the observed behaviors and reveals the possible associations between these behaviors through the calculation of observed and expected probabilities (Bakeman and Quera, 2011). Specifically, the data used for this analysis were type II: event-based and concurrent (Bakeman, 1978). To perform data analysis, we used GSEQ v. 5.1 computer program (Bakeman and Quera, 2011).

Starting from a certain behavior *(criterion behavior)*, selected based on the objectives of the study, it allows us to know what other behaviors *(conditioned behaviors)* precede and follow it, through retrospective (lags −1, −2, −3, etc.) and prospective (lags +1, +2, +3, etc.) lag sequential analyses. The results obtained are adjusted residuals, which show the likelihood of appearing of each conditioned behavior in every negative and positive lag that is studied. Depending on the adjusted residual obtained, we can assess if the likelihood of appearing is higher than the effects of chance, with a significance level of *p*< 0.05 (adjusted residual > 1.96). The concurrence of behaviors can be studied, too, considering the adjusted residuals obtained in lag 0.

We first used this analytical technique to study if educators’ instrumental action followed specific behavioral patterns, so the behaviors under this particular subdimension were included in the analysis (Table 2).

**TABLE 2:** Behaviors under the instrumental action (A2) subdimension of the field format.

A2 – Instrumental action	A200 – None
	A201 – Opens/closes door, cupboard
	A202 – Passes from top
	A203 – Picks up materials, toys
	A204 – Tidies up materials, toys
	A205 – Transports materials, toys
	A206 – Tidies up spaces
	A207 – Dresses dolls
	A208 – Offers materials, toys
	A209 – Looks for materials, toys
	A210 – Gives material, toy to a child
	A211 – Distributes to children
	A212 – Accompanies complex activity
	A213 – Throws away (to the paper bin)
	A214 – Folds (blanket, clothes)
	A215 – Moves/drags recipients with toys on the floor
	A216 – Other
	A217 – Accepts toy from child
	A218 – Holds materials, toys

Then, we calculated the concurrences between the instrumental actions that were part of the behavioral patterns and the relational behaviors under the emblems, illustrators, and regulators subdimensions (see Table 3).

**TABLE 3:** Behaviors under the emblems (P9), illustrators (P10), and regulators (P11) subdimensions of the field format.

P9 – Emblems	P900 – No emblem
	P901 – Points/shows
	P902 – Opens hand to ask for
	P903 – Tilts her head
	P904 – Announces her departure
	P905 – “I see that”
	P906 – Strokes/looks after toy
	P907 – Strategic position
	P908 – Waits, gives time
	P909 – Verifies, does it again
	P910 – Overtakes hand (secures)
	P911 – Asks by lifting head
	P912 – Asks by opening hands
	P913 – Raises hand (says hi)
P10 – Illustrators	P1000 – No illustrator
	P1001 – Nods
	P1002 – Shakes her head (“no”)
	P1003 – Tilts her head
	P1004 – Arms crossed/centripetal
	P1005 – Arms open/centrifugal
	P1006 – Changes gaze to another target
	P1007 – Focused gaze
	P1008 – Mediates with arm (in conflict)
P11 – Regulators	P1100 – No regulator
	P1101 – Marks beginning
	P1102 – Her action is before child’s next
	P1103 – Makes child do
	P1104 – Lets child do, and she continues her action
	P1105 – Lets child do, and she stops her action
	P1106 – Follows child’s action
	P1107 – Marks ending
	P1108 – Accepts child’s “no”
	P1109 – Last touch

## Results

The first aim of this study was to discover behavioral patterns in the educators’ instrumental action. Therefore, all the behaviors under the instrumental action subdimension (A2) included in the observation instrument, 19 in total, were first subjected to lag sequential analysis. All of them were selected as criterion and conditioned behaviors, as we wanted to discover the relationship between them. We conducted retrospective and prospective lag sequential analyses (lags −5 to +5) to obtain information about what happens before and after each of the criterion behaviors. The same analyses were done with both educators’ recordings. Due to the complexity of educators’ behavior and the molecularity of our observation instrument, we found that some behaviors had significant results in adjacent lags. Therefore, patterns of interest were highlighted in the following tables, according to the aims of the study.

**FIGURE 1:**
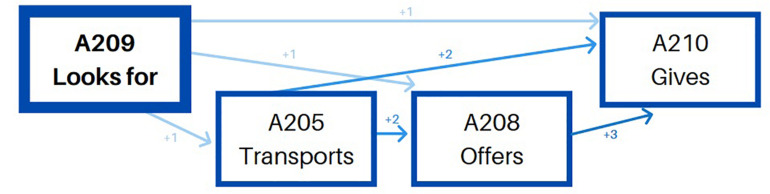
Educator 1. “Looks for” pattern.

For educator 1, we found two behavioral patterns: “looks for a toy/material” and “transports toy/material.” The first pattern – looks for toy/material – (see Table 4) is formed by four behaviors that can shape four different pattern combinations. It always starts with her looking for a toy or material and finishes by giving it to a child. It can be a simple succession of just these behaviors or become more complex, in case she also transports, offers, or performs both behaviors, as shown in Figure 1.

**TABLE 4:** Significant adjusted residuals of the lag sequential analysis for educator 1.

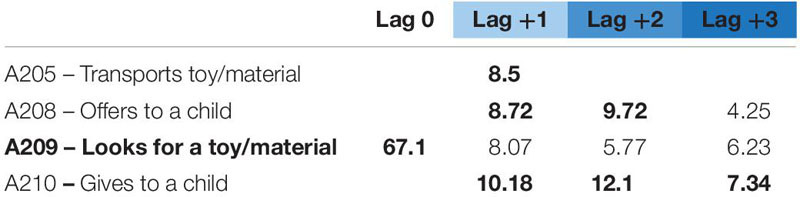

*“Looks for toy/material” pattern.*

**FIGURE 2:**
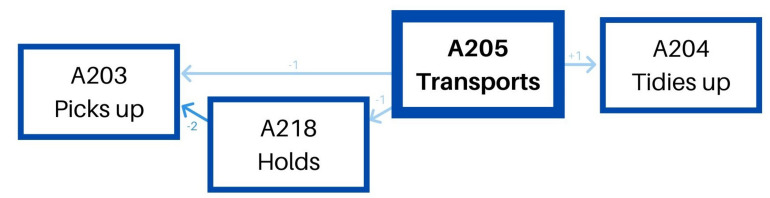
Educator 1. “Transports” pattern.

The second pattern – transports toy/material – (see Table 5) is less versatile. Before transporting a toy, she first picks it up, and she can also hold it for a while or not. Then, she transports it and tidies up on its final position systematically (Figure 2).

**TABLE 5:** Significant adjusted residuals of the lag sequential analysis for educator 1.

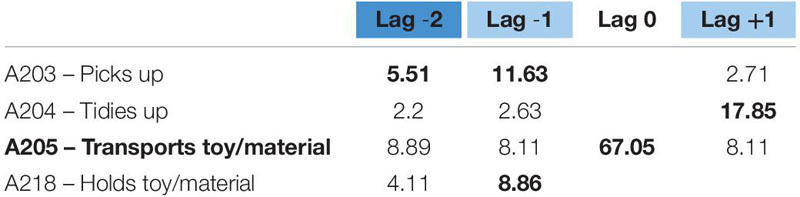

*“Transports toy/material” pattern.*

**FIGURE 3:**
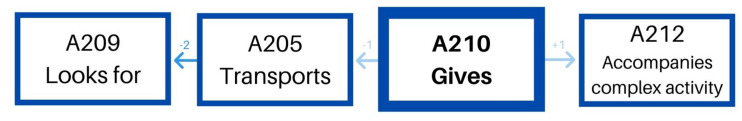
Educator 2. “Gives” pattern.

For educator 2, we found another two behavioral patterns: “gives toy/material to a child” and “transports toy/material.” The first pattern – gives toy/material – (see Table 6) is a very systematic succession of four different behaviors, as shown in Figure 3. Before giving a toy, she looks for it and later transports it to another area of the classroom. Then, she gives it to a child, and, finally, she accompanies a complex activity related to the object involved in the action (book reading or puzzle-solving).

**TABLE 6:** Significant adjusted residuals of the lag sequential analysis for educator 2.

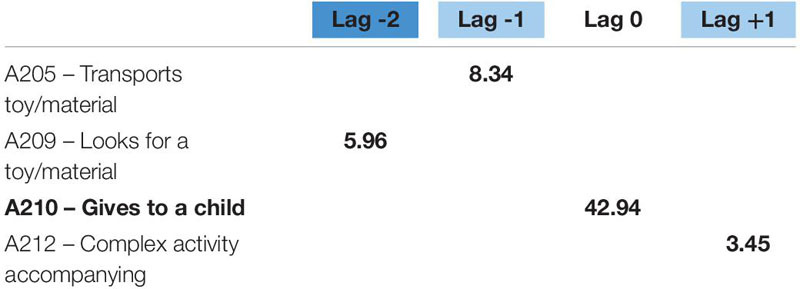

*“Gives toy/material” pattern.*

**FIGURE 4:**
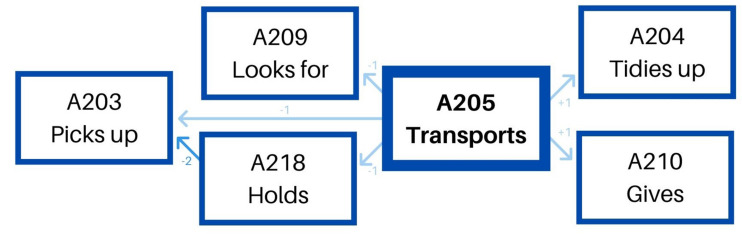
Educator 2. “Transports” pattern.

The second pattern – transports toy/material – (see Table 7) has two different options. Before transporting a toy, on the one hand, she can pick it up, and after she transports it, she can tidy it up on its final position. In this case, between the moment she picks it up and transports it, she can also hold it for some time. On the other hand, she can previously look for a specific toy, transport it, and give it to a child once in the final area of the classroom. This last option has a similar shape to the previously explained pattern: gives toy/material. Both options are included in the same pattern, as shown in Figure 4.

**TABLE 7:** Significant adjusted residuals of the lag sequential analysis for educator 2.

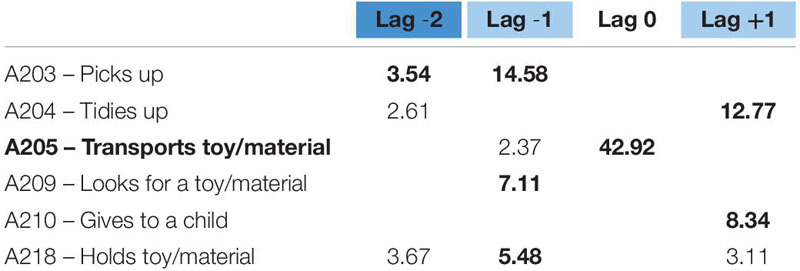

*“Transports toy/material” pattern.*

All in all, we found four different behavioral patterns when studying the instrumental dimension of the educators’ behavior. One of them is common for both educators – transports toy/material – even if it is shaped quite differently by each of them. The second behavioral pattern, on the contrary, is particular for each of the educators.

Once the behavioral patterns for the instrumental dimension were analyzed, we wanted to study the relational dimension to discover their specificities. Having this in mind, we analyzed the lag 0 or concurrence of the instrumental behaviors that constitute the behavioral patterns we had obtained for the instrumental dimension criterion behaviors – and some of the behaviors under the subdimensions included in the relational dimension. Specifically, we analyzed the concurrence with the emblems, illustrators, and regulators performed by the educators – conditioned behaviors.

The same analyses were carried out with both educators’ recordings. The significant results found for each of them are shown in Tables 8, 9.

**TABLE 8:** Concurrence of educators’ instrumental and relational behaviors.

	**P9 Emblems**	**P10 Illustrators**	**P11 Regulators**
A203 – Picks up	P900 – None 8.61	P1000 – None 6.99	P1100 – None 5.7
A204 – Tidies up	P900 – None 8.07	P1000 – None 7.22	P1109 – Last touch 7.45
A205 – Transports	P900 – None 5.73	P1000 – None 5.6	P1100 – None 4.73
A208 – Offers	P901 – Points/shows 15.1	–	P1103 – Makes child do 4.25
A209 – Looks for	P900 – None 3.76	P1000 – None 2.61	P1100 – None 2.42
A210 – Gives	P900 – None 3.12	P1000 – None 3.03	P1101 – Marks beginning 7.84
			P1103 – Makes child do 10.69
A218 – Holds	P906 – Strokes/looks after toy 12.7	P1003 – Tilts her head 2.67	P1108 – Accepts child’s “no” 2.17

*Educator 1.*

**TABLE 9:** Concurrence of educators’ instrumental and relational behaviors.

	**P9 Emblems**	**P10 Illustrators**	**P11 Regulators**
A203 – Picks up	P900 – None 4.23	P1000 – None 3.84	P1100 – None 2.77
A204 – Tidies up	P900 – None 5.7	P1000 – None 5.22	P1100 – None 3.03
A205 – Transports	P900 – None 3.15	P1000 – None 3.84	P1100 – None 3.34
A209 – Looks for	P900 – None 2.89	P1000 – None 2.22	P1100 – None 2.7
A210 – Gives	P907 – Strategic position 2.65	–	–
A212 – Accompanies	–	–	P1102 – Her action is ahead child’s next 15.5
A218 – Holds	P906 – Strokes/looks after toy 3.94 P911 – Asks with head 3.97	P1008 – Mediates w/arm 4.44	P1105 – Lets child do and she stops her action 4.36

*Educator 2.*

The adjusted residuals obtained in lag 0 confirm the existence of significant relationships between the instrumental and the relational dimensions of educators’ behavior.

## Discussion

The obtained results meet the objectives proposed in this study. Moreover, they present a very detailed picture of the studied educators’ behavior while they are accompanying children’s free play.

The first objective was to discover if the educators’ instrumental action (Wallon, 1980) follows a systematic succession of behaviors: different but precise physical actions related to children’s free play accompanying. As we had hypothesized, we found behavioral patterns when studying the instrumental dimension of their educational activity: two for each of them. The strong results obtained demonstrate that the instrumental dimension of their educational activity – what they do while children are playing – is systematic and well-established. The order in which their instrumental behaviors are carried out is not an effect of chance, but they act in a very particular way, meticulously following an established behavioral sequence or *choreography*.

The results concerning the second objective of the study indicate that, together with acting in an organized way, the relational dimension of their educational activity is specific for each of the instrumental behaviors. The findings indicate that the relational behaviors support educators’ instrumental action if they target children, for example, when they help children who are facing a complex task (A212) when they hold an object (A218) while tidying up to follow children’s cues, offering (A208) and giving (A210) new materials if they seem bored, or they ask for something specific. Those instrumental behaviors are mediated by some kinetic elements – emblems (Ekman, 1976), illustrators, and regulators (Ekman and Friesen, 1969) – which complete educators’ instrumental action. Those actions are combined with other ones – tidying up (A204), picking up toys from the floor (A203), looking for them in the cupboards (A209), and transporting materials (A205) – directed to objects, materials, or the classroom layout in general. The results show that when their instrumental action is directed to objects or materials instead of children, there are less relational behaviors implied.

**FIGURE 5:**
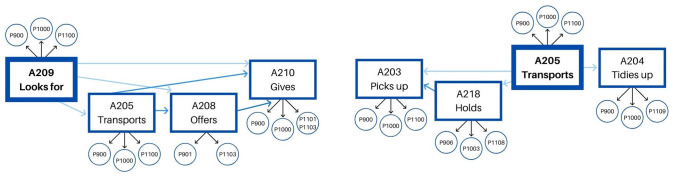
Educator 1. Instrumental patterns and their relational value.

**FIGURE 6:**
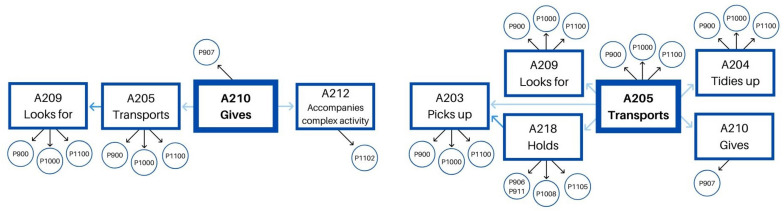
Educator 2. Instrumental patterns and their relational value.

We can, therefore, demonstrate the existence of free play accompanying action patterns, formed by a masterly and differentiated combination of actions that include instrumental and relational dimensions (Figures 5, 6). Therefore, together with performing systematic instrumental patterns, the relational value is specific to each of the behaviors that constitute them.

The great value of the free play accompanying action patterns lies in the precise and meticulous adaptation of the observed educators’ actions to each child or the particular circumstances of an everyday life moment as children’s free play episodes. This way, the hypothesis for the third objective of the study is confirmed: it is possible to distinguish three different intervention levels, depending on the intensity of educators’ behavior toward young children: *direct*, *indirect*, and *no intervention* or *presence*. First, we just found *direct intervention* when educator 2 helps children who are facing a complex task. In this case, her action is even before children’s next, as regulator P1102 shows. So, when children ask for her support to solve a challenging activity, her role is to accompany this activity and provide meaningful feedback directly, so they can ultimately learn to solve that challenge on their own. The *indirect intervention* appears when educators’ action is directed to children, but they just support children’s own activity from an outsider point of view. This happens when they offer, give, or hold materials that children need or may need. The instrumental behaviors’ evident relational value proofs educators’ support, but, at the same time, they do not disrupt children’s initiative. Finally, when they tidy up, pick up, transport, and look for objects, there is *no intervention*, but they are present, trying to keep the playing area tidy, so children’s inherent interest is always renovated. Therefore, the intervention level of the observed educators’ educational activity is defined by the combination of their instrumental behaviors and their specific relational value and is adaptive to each child and the circumstances of the moment.

As a result of these systematic behavioral patterns, formed by actions translated into different intervention levels, we conclude that the observed educators ensure that the three basic psychological needs proposed by self-determination theory are satisfied. The stability of the educators’ instrumental action provides children with a sense of security and relatedness (Deci and Ryan, 2000); the environment where they are playing is predictable and familiar, so they feel safe and open to play (Kálló and Balog, 2013). At the same time, through the adaptation of the relational dimension of their actions, they make sure that each child’s play needs are satisfied. Therefore, children will always be open to exploring, so they can live their own competence (White, 1959; Pikler, 2018) in each developmental stage and at any moment. Finally, educators never suggest games directly, but they indirectly accompany children’s playful activity, and even when they support children who are facing a complex task, it is children who ask for the educator’s help. Therefore, their educational activity consists of ensuring that children’s actions are always a result of their internal needs and their capacities. They are self-endorsed, so they promote children’s autonomy (Deci and Ryan, 2000).

The adaptation of the educators’ actions to the circumstances of each moment can be seen as facilitating so that children can be focused on their own activity. Consequently, children’s activity will potentially evolve from subjective to more objective actions toward the external world (Wallon, 1980). The functional integration of those discoveries results in more complex activities and thoughts (Wallon, 2008). So, children’s action toward the external world permits the construction of their internal world; it enables their psychological construction. Like this, the emergence of the self will come naturally and, together with that, a self-determined behavior. The environment enables child development, but the child is the protagonist of his or her own development.

The intentional and adaptive early-childhood-oriented educational behavior that characterizes the observed educators serves as a delicate and specific combination of instrumental and relational dimensions that provides the physical and psychological conditions (Wallon, 1985) for children’s playful activity to develop thoroughly. This is only possible due to the value given to everyday moments’ observation from this educational approach, which permits us to get to know every single detail about each child and the group in general (Tardos, 2018b). The regular and permanent observations and the educational work behind them ensure the adjustment of the educational activity to the specific situation, so it is coherent for children (Mózes, 2016): they guarantee an understanding and understandable environment around every child (Falk, 2018b), built in each particular moment through the child’s autonomous activity and the adaptation of the educator’s educational activity to it. As Mózes (2016) mentioned, the educator’s role while children are playing on their own can be translated into a psychological positioning toward them. Hence, educators interact with children whenever they need it and just when they do, always adapting their interventions to the child and situation, providing proper autonomy support.

In conclusion, our results add to the previous literature suggesting that Pikler–Lóczy educational approach is far from ideological but, instead, is built in everyday life moments, through the educators’ intentional, deconstructed, and meticulously designed educational activity, where children are not just said to be the protagonists of their development, but they actually are. The intensity of the observed educators’ intervention is inversely proportional to children’s capacities. Thus, to the *direct intervention* toward children, commonly found when studying daily care routines (Belasko et al., 2019; Belza et al., 2019a, b) or when they attend to specific demands of children during free play, we can now add the *indirect intervention* and *no intervention*. The exquisite management of the intervention levels by the observed educators evidences the coherence of this educational approach with self-determination theory and, especially, with the three basic psychological needs presented through it: autonomy, competence, and relatedness. This study contributes to the idea that Pikler–Lóczy education provides an autonomy-supportive environment directed to young children and applied in a collective institution as a nursery school.

### Limitations and Future Research

Due to the complexity of Pikler educators’ educational activity and the magnitude of the obtained results, we could not cover all aspects of the studied dimensions. In a future study, it would be of interest to deepen in the rest of the subdimensions included in the relational dimension of the field format (P1–P8) to discover more details of the relational aspect of each of their instrumental actions. Furthermore, future research should go into detail about the three intervention levels found in Pikler educators: direct intervention, indirect intervention, and no intervention or presence. An in-depth study based on the observational data already collected could shed light on the behaviors that cause and inhibit the appearance of each of the intervention levels. Finally, studying the verbal elements that mediate the educators’ indirect and direct interventions displayed while they accompany children’s free play would complement the obtained results in a very meaningful way.

## Data Availability Statement

The original contributions presented in the study are included in the article/supplementary material, further inquiries can be directed to the corresponding author.

## Ethics Statement

The studies involving human participants were reviewed and approved by the University of the Basque Country Ethics Committee on research involving human beings. Written informed consent to participate in this study was provided by the participants’ legal guardian/next of kin.

## Author Contributions

JS codified the videos, developed statistical analyses, documented, designed, and wrote the manuscript. EH collected the data, developed the field format, and contributed to the conceptual structure of the research work. MTA contributed to the methodological structure of the study and offered guidance on the data analysis. All authors provided approval of the final version of the manuscript to be published.

## Conflict of Interest

The authors declare that the research was conducted in the absence of any commercial or financial relationships that could be construed as a potential conflict of interest.

## References

[B1] Anguera M. T. (2001). Cómo apresar las competencias del bebé mediante una aplicación de la metodología observacional [How to catch the baby’s skills by applying observational methodology]. *Context. Educ.* 4 13–34. 10.18172/con.484

[B2] Anguera M. T. (2003). “La observación [Observation],” in *Evaluación Psicológica. Concepto, Proceso y Aplicación De Las Áreas Del Desarrollo Y De La Inteligencia*, ed. Moreno Rosset C. (Madrid: Sanz y Torres), 271–308.

[B3] Anguera M. T. (2010). Posibilidades y relevancia de la observación sistemática por el profesional de la psicología [Possibilities and relevance of systematic observation by the psychology professional]. *Papeles del Psicólogo* 31 122–130.

[B4] Anguera M. T. Blanco-Villaseñor A. Hernández-Mendo A. Losada J. L. (2011). Diseños observacionales: ajuste y aplicación en psicología del deporte [Observational designs: adjustment and application in sports psychology]. *Cuadernos Psicol. Deport.* 11 63–76.

[B5] Anguera M. T. Izquierdo C. (2006). “Methodological approaches in human communication. From complexity of situation to data analysis,” in *From Communication to Presence. Cognition, Emotions and Culture towards the Ultimate Communicative Experience*, eds Riva G. Anguera M. T. Wiederhold B. K. Mantovani F. (Amsterdam: IOS Press), 203–222.

[B6] Anguera M. T. Magnusson M. Jonsson G. (2007). Instrumentos no estandar: planteamiento, desarrollo y posibilidades [Non-standard instruments: approach, development and possibilities]. *Avances Med.* 5 63–82.

[B7] Bakeman R. (1978). “Untangling streams of behaviour: Sequential analysis of observation data,” in *Observing Behaviour, Vol. 2: Data Collection and Analysis Methods*, ed. Sackett G. P. (Baltimore, MD: University of Park Press), 63–78.

[B8] Bakeman R. Gottman J. M. (1997). *Observing Interaction. An Introduction To Sequential Analysis.* Cambridge: Cambridge University Press.

[B9] Bakeman R. Quera V. (2011). *Sequential Analysis And Observational Methods For The Behavioral Sciences.* Cambridge: Cambridge University Press.

[B10] Belasko M. Herrán E. Anguera M. T. (2019). Dressing toddlers at the Emmi Pikler nursery school in Budapest: educator instrumental behavioral pattern. *Eur. Early Childh. Educ. Res. J.* 27 872–887. 10.1080/1350293X.2019.1678928

[B11] Belza H. Herrán E. Anguera M. T. (2019a). Early childhood, breakfast, and related tools: analysis of adults’ function as mediators. *Eur. J. Psychol. Educ.* 10.1007/s10212-019-00438-4

[B12] Belza H. Herrán E. Anguera M. T. (2019b). Early childhood education and cultural learning: systematic observation of the behaviour of a educator at the Emmi Pikler nursery school during breakfast. *Infan. Aprend.* 42 128–178. 10.1080/02103702.2018.1553268

[B13] Bernier A. Carlson S. M. Whipple N. (2010). From external regulation to self-regulation: Early parenting precursors of young children’s executive functioning. *Child. Dev.* 81 326–339. 10.1111/j.1467-8624.2009.01397.x 20331670

[B14] David M. Appell G. (1986). *La Educación del Niño de 0 a 3 Años: Experiencia del Instituto Lóczy* [The Education of the Child From 0 to 3 Years: Experience Madrid: Narcea.

[B15] David M. Appell G. (2010). *Lóczy. Una Insólita Atención Personal [Lóczy: An Extraordinary Personal Attention].* Octaedro: Barcelona.

[B16] Deci E. L. (1975). *Intrinsic Motivation.* New York, NY: Plenum.

[B17] Deci E. L. (1995). *Why We Do What We Do: Understanding Self-Motivation.* New York, NY: Penguins Books.

[B18] Deci E. L. Ryan R. M. (1985). *Intrinsic Motivation And Self-Determination In Human Behavior.* New York, NY: Plenum.

[B19] Deci E. L. Ryan R. M. (1991). “A motivational approach to self: Integration in personality,” in *Nebraska Symposium on Motivation: Vol. Maternal Autonomy Support 123338. Perspectives on Motivation*, ed. Dienstbier R. (Lincoln: University of Nebraska Press), 237–288. 2130258

[B20] Deci E. L. Ryan R. M. (2000). The “what” and” why” of goal pursuits: human needs and the self-determination of behavior. *Psychol. Inq.* 11 227–268.

[B21] Deci E. L. Schwartz A. J. Sheinman L. Ryan R. M. (1981). An instrument to assess adults’ orientations toward control versus autonomy with children: reflections on intrinsic motivation and perceived competence. *J. Educ. Psychol.* 73 642–650. 10.1037/0022-0663.73.5.642

[B22] Ekman P. (1976). Movements with precise meanings. *Nonverb. Commun.* 26 14–26. 10.1111/j.1460-2466.1976.tb01898.x

[B23] Ekman P. Friesen W. V. (1969). The repertoire of nonverbal behavior: categories, origins, usage and coding. *Semiótica* 1 49–98.

[B24] European Commission (2019). *Key Data on Early Childhood Education and Care in Europe—2019 Edition. Eurydice Report.* Luxembourg: Publications Office of the European Union.

[B25] Falk J. (2018a). “El hecho consciente en lugar de la instintividad. Sustitución eficaz de la relación madre-niño en la casa cuna [Conscious act instead of instinct. Effective substitution of the mother-child relationship in the foster home],” in *Claves de la Educación Pikler-Lóczy. Compilación de 20 Artículos Escrito spor sus Creadoras*, ed. Herrán E. (Budapest: Asociación Pikler-Lóczy de Hungría), 179–188.

[B26] Falk J. (2018b). “Los fundamentos de una verdadera autonomía en el niño pequeño [The foundations of true autonomy in the young child],” in *Claves de la Educación Pikler-Lóczy. Compilación de 20 Artículos Escrito spor sus Creadoras*, ed. Herrán E. (Budapest: Asociación Pikler-Lóczy de Hungría), 89.114.

[B27] Hall E. (1966). *The Hidden Dimension.* Garden City, NY: Doubleday.

[B28] Hernández Mendo A. López J. A. Castellano Paulis J. Morales Sánchez V. Pastrana Brincoles J. L. (2012). HOISAN 1.2: Programa informático para uso en Metodología Observacional [HOISAN 1.2: computer program for use in observational methodology]. *Cuadernos Psicol. Deport.* 12 55–78. 10.4321/s1578-84232012000100006 31832788

[B29] Hernández-Mendo A. Castellano J. Camerino O. Jonsson G. Blanco-Villaseñor Á Lopes A. (2014). Programas informáticos de registro, control de calidad del dato, y análisis de datos [Computer software for recording, data quality control and data analysis]. *Rev. Psicol. Deport.* 23 111–121.

[B30] Herrán E. (2013). La educación Pikler-Lóczy. Cuando educar empieza por cuidar [Pikler-Lóczy education: when education starts with caregiving]. *Rev. Latinoam. Educ. Infant.* 2 37–56.

[B31] Hewes J. (2014). Seeking balance in motion: the role of spontaneous free play in promoting social and emotional health in early childhood care and education. *Children* 1 280–301. 10.3390/children1030280 27417480 PMC4928743

[B32] Hirsh-Pasek K. Golinkoff R. M. Berk L. E. Singer D. (2009). *A Mandate for Playful Learning in Preschool: Applying the Scientific Evidence.* New York, NY: Oxford University Press.

[B33] Joussemet M. Koestner R. Lekes N. Landry R. (2005). A longitudinal study of the relationship of maternal autonomy support to children’s adjustment and achievement in school. *J. Pers.* 73 1215–1236. 10.1111/j.1467-6494.2005.00347.x 16138871

[B34] Kálló E. Balog G. (2013). *Los Orígenes Del Juego Libre [The Origins Of Free Play].* Budapest: Magyarországi Pikler-Lóczy Társasag.

[B35] Kelemen J. (2016). El trayecto profesional de una cuidadora pikleriana [The profesional career of a Pikler educator]. *RELAdEI Rev. Latinoam. Educ. Infant.* 5 36–40.

[B36] Kochanska G. Coy K. C. Murray K. T. (2001). The development of self-regulation in the first four years of life. *Child Dev.* 72 1091–1111. 10.1111/1467-8624.00336 11480936

[B37] Krippendorff K. (2013). *Content Analysis. An Introduction To Its Methodology*, 3rd Edn, Thousand Oaks, CA: Sage.

[B38] Mózes E. (2016). La observación en la Pedagogía Pikler [Observation in Pikler Pedagogy]. *Rev. Latinoamericana Educ. Infant.* 5 27–35.

[B39] Niemiec C. P. Ryan R. M. (2009). Autonomy, competence, and relatedness in the classroom: Applying self-determination theory to educational practice. *Theor. Res. Educ.* 7 133–144. 10.1177/1477878509104318

[B40] Ormaza A. (2016). “Observación de la actividad autónoma infantil en la escuela infantil Pikler-Lóczy desde la metodología observacional. Un estudio exploratorio [Observation of children’s autonomous activity in the Pikler-Lóczy nursery school from the observational methodology. An exploratory study],” in *XXIII. Jornadas de Investigación en Psicodidáctica*, eds Rodriguez A. Romero A. Ros I. (Bilbao: UPV-EHU), 3–23.

[B41] Pavlov I. P. (1960). *Conditioned Reflexes: An Investigation of the Physiological Activity of the Cerebral Cortex.* New York, NY: Dover. 10.5214/ans.0972-7531.1017309 PMC4116985 25205891

[B42] Pikler E. (1940). *Mit Tud Már A Baba? What Does Your Baby Already Know?.* Budapest: Cserépfalvi.

[B43] Pikler E. (1968). Some contributions to the study of the gross motor development of children. *J. Genet. Psychol.* 113 27–39. 10.1080/00221325.1968.10533806 5708789

[B44] Pikler E. (1969). *Moverse en Libertad: Desarrollo de la Motricidad Global [Moving Around Freely: Developing Global Motor Skills].* Madrid: Narcea.

[B45] Pikler E. (1988). *Laßt Mir Zeit: Die Selbständige Bewegungsentwicklung des Kindes bis Zum Freien Gehen [Give Me Time: The Child’s Independent Movement Development Until Free Walking].* München: Pflaum Verlaug.

[B46] Pikler E. (1998). Importancia del movimiento en el desarrollo de la personalidad. Iniciativa-competencia [Importance of movement in the development of personality. Initiative-competence]. *La Hamaca* 9 31–42.

[B47] Pikler E. (2018). “La competencia del bebé [The baby’s competence],” in *Claves de la educación Pikler-Lóczy. Compilación de 20 Artículos Escritos por sus Creadoras*, ed. Herrán E. (Budapest: Asociación Pikler-Lóczy de Hungría), 59–71.

[B48] Pikler E. Tardos A. (1968). Megfigyelések a csecsemõ nagymozgásos aktivitásának alakulásáról az oldalrafordulástól a biztos járásig [Observations on the development of the infant’s high physical activity from turning to safe walking]. *Magyar Pszichol. Szemle* 25 69–86.

[B49] Portell M. Anguera M. T. Chacón S. Sanduvete S. (2015). Guidelines for reporting evaluations based on observational methodology. *Psicothema* 27 283–289. 10.7334/psicothema2014.276 26260937

[B50] Poyatos F. (1986). Enfoque integrativo de los componentes verbales y no verbales de la interacción y sus procesos y problemas de codificación [Integrative approach of the verbal and non-verbal components of the interaction and its processes and coding problems]. *Anuario psicol.* 34 125–155.

[B51] Ryan R. M. Deci E. L. (2000). Intrinsic and extrinsic motivations: classic definitions and new directions. *Contemp. Educ. Psychol.* 25 54–67. 10.1006/ceps.1999.1020 10620381

[B52] Ryan R. M. Deci E. L. (2011). “A self-determination theory perspective on social, institutional, cultural, and economic supports for autonomy and their importance for well-being,” in *Human Autonomy In Cross-Cultural Context*, eds Chirkov V. Ryan R. Sheldon K. (Dordrecht: Springer), 45–64. 10.1007/978-90-481-9667-8_3

[B53] Ryan R. M. La Guardia J. G. (2000). “What is being optimized over development?: A self-determination theory perspective on basic psychological needs across the life span,” in *Dialogues on Psychology and Aging*, eds Qualls S. Abeles R. (Washington, DC: American Psychological Association), 145–172. 10.1037/10363-008

[B54] Sánchez-Algarra P. Anguera M. T. (2013). Qualitative/quantitative integration in the inductive observational study of interactive behaviour: impact of recording and coding among predominating perspectives. *Qual & Quant* 47 1237–1257. 10.1007/s11135-012-9764-6

[B55] Schegloff E. (2000). On granularity. *Annu. Rev. Sociol.* 26 715–720. 10.1146/annurev.soc.26.1.715

[B56] Szöke A. (2016). Autónomo, pero no abandonado a su suerte. El apoyo de la actividad autónoma en la escuela infantil 0-3 [Autonomous, but not abandoned to his fate. Support of autonomous activity in nursery school 0-3]. *Rev. Latinoamericana Educ. Infant.* 5 41–46.

[B57] Tardos A. (1964). Megfigyelések a környezetváltozásnak a csecsemõk játéktevékenységére gyakorolt hatásáról. *Pszichol. Tanulmányok* 6 273–287.

[B58] Tardos A. (1967). A 3-12 hónapos csecsemõ optikus és taktilmotoros explorációs viselkedése [Optical and tactile motor exploratory behavior of an infant 3-12 months of age]. *Magyar Pszichol. Szemle* 24 57–70.

[B59] Tardos A. (1972). A csecsemõ mozgásfejlõdése során megnyilvánuló tanulási folyamatok egyes sajátosságai [Some peculiarities of the learning processes manifested during the developmental movement of the infant]. *Pszichol. Tanulmányok* 13 281–287.

[B60] Tardos A. (2010). The researching infant. *Signal* 18 9–14.

[B61] Tardos A. (2014). *El Adulto y el juego del niño [The Adult and The Child’s Play].* Barcelona: Octaedro.

[B62] Tardos A. (2018a). “Dejemos al niño también jugar solo [Let’s leave the child play alone too],” in *Claves de la Educación Pikler-Lóczy. Compilación de 20 Artículos Escritos por sus Creadoras*, ed. Herrán E. (Budapest: Asociación Pikler-Lóczy de Hungría), 245–258.

[B63] Tardos A. (2018b). “El papel de la observación en el trabajo educativo [The role of observation in the educational work],” in *Claves de la educación Pikler-Lóczy. Compilación de 20 Artículos Escritos por sus Creadoras*, ed. Herrán E. (Budapest: Asociación Pikler-Lóczy de Hungría), 115–124.

[B64] Tardos A. David M. (2018). “Del valor de la actividad libre en la elaboración del yo [About the value of free activity in the elaboration of the self],” in *Claves de la Educación Pikler-Lóczy. Compilación de 20 Artículos Escritos por sus Creadoras*, ed. Herrán E. (Budapest: Asociación Pikler-Lóczy de Hungría), 347–376.

[B65] Tardos A. Szanto-Feder A. (2018). “Qué es la autonomía desde la primera edad? [What’s autonomy from the early ages?],” in *Claves de la Educación Pikler-Lóczy. Compilación de 20 Artículos Escritos por sus Creadoras*, ed. Herrán E. (Budapest: Asociación Pikler-Lóczy de Hungría), 73–87.

[B66] Tomasello M. (2000). *The Cultural Origins of Human Cognition.* Cambridge, MA: Harvard University Press.

[B67] Vygotsky L. S. (2000). *El Desarrollo de los Procesos Psicológicos Superiores [Development of Higher Psychological Processes].* Crítica: Barcelona.

[B68] Wallon H. (1974). *Del Acto al Pensamiento [From Act To Thought].* Psique: Buenos Aires.

[B69] Wallon H. (1980). *Psicología del niño. Una Comprensión Dialéctica Del Desarrollo Infantil [Child Psychology. A Dialectical Understanding of Child Development].* Madrid: Pablo del Río.

[B70] Wallon H. (1985). *La Vida Mental [Mental Life].* Crítica: Barcelona.

[B71] Wallon H. (2008). *La Evolución Psicológica del Niño [The Psychological Evolution of The Child].* Crítica: Barcelona.

[B72] Whipple N. Bernier A. Mageau G. A. (2011). A dimensional approach to maternal attachment state of mind: relations to maternal sensitivity and maternal autonomy support. *Dev. Psychol.* 47 396–403. 10.1037/a0021310 21171748

[B73] White R. W. (1959). Motivation reconsidered: the concept of competence. *Psychol. Rev.* 66 297–333. 10.1037/h0040934 13844397

[B74] Yogman M. Garner A. Hutchinson J. Hirsh-Pasek K. Golinkoff R. M. (2018). The power of play: a pediatric role in enhancing development in young children. *Pediatrics* 142:e20182058. 10.1542/peds.2018-2058 30126932

